# Experimental Research on Schema Modes: Guidelines and Research Agenda

**DOI:** 10.1007/s11920-026-01663-7

**Published:** 2026-02-09

**Authors:** Julie Krans, Samuel Hilgefort, Jill Lobbestael

**Affiliations:** 1 Behavioural Science Institute, Radboud University, Nijmegen, the Netherlands; 2 Pro Persona Research, Nijmegen, the Netherlands; 3 Faculty of Psychology and Neuroscience, Department of Clinical Psychological Science, Maastricht University, Maastricht, the Netherlands

**Keywords:** Schema therapy, Schema modes, Experimental, Emotion induction, Personality disorders

## Abstract

**Purpose of Review:**

Schema therapy is an evidence-based treatment for personality disorders (PDs), with ‘schema modes’ being a core theoretical and clinical concept. Despite their centrality, empirical research into the mechanisms of schema mode activation remains limited. This paper reviews the available research on schema mode activation and provides methodological guidelines and a research agenda to stimulate experimental research on schema modes.

**Recent Findings:**

We identified five published studies that experimentally induced schema modes using a variety of procedures (e.g., stress interviews, film clips, and art/drama therapy), showing diverse but generally significant effects on mode activation. Their findings underscore the need for validated and replicable experimental paradigms, as effects were highly dependent on sample characteristics, procedure-specific variability, and discrepancies between therapist- and patient ratings of schema mode activation. Building on insights from emotion induction research, we propose two procedures for experimental schema mode induction: guided autobiographical recall/scripted imagery and stimulus-based experimental triggers. We outline best practices, challenges, and recommendations for designing and validating such procedures. A future research agenda is presented, focusing on identifying conditions that activate schema modes and examining their effects on information processing and behavior.

**Summary:**

By developing validated experimental tools and adopting open science practices, we aim to stimulate further experimental research to empirically test schema therapy’s theoretical premises and clarify the role of schema modes in PDs.

## Introduction

Personality disorders (PDs) affect daily life through problems in identity, interpersonal relations and self-management [1]. Recent estimates of the prevalence of PDs globally are 3.8%, 2.8%, and 5.0% for cluster A, B, and C, respectively [2]. The societal costs of PDs, especially due to productivity loss, are substantial [3]. Schema therapy [4] is an effective treatment for PDs [5–7] and borderline PD in particular [8], with preliminary findings showing superiority in symptom reduction, suicidality, and drop-out rates compared to other evidence-based treatments for borderline PD [9]. The theory of schema therapy proposes that unmet basic emotional needs in childhood (e.g., safety, self-expression) can result in early maladaptive schemas about the self in relation to others and the world [4]. When these schemas are triggered, they can result in transient emotional states and coping responses called ‘schema modes’. Which schema mode occurs after a schema is triggered is proposed to depend on the individual’s coping style: resignation (adopting the schema as completely true and behaving in line with it), avoidance (avoiding schema activation through behavioral strategies, such as self-soothing), and inversion (adopting the opposite view of the schema and behaving in an opposite way to disprove it) [10]. The concept of schema modes was introduced in schema theory to understand and label rapid shifts in emotions, cognitions and behaviors characteristic of PDs [4]. For instance, rapid transitions between overwhelming child-like emotions (i.e., Vulnerable Child mode) and feeling numb and detached (i.e., Detached Protector mode) exemplify the emotional and behavioral volatility in borderline PD [11]. Despite its importance in the theoretical foundations and clinical application of schema therapy, schema modes have not received much empirical attention, and researchers have been calling out for more studies [11–13] (see also Pilkington et al. [14] for a Delphi study listing the research priorities for schema therapy). 

The schema mode concept has been validated in studies that show correlations between self-report measures of schema modes in different PDs consistent with theoretical expectations [12, 13, 15, 16]; for example, the Detached Protector, Punitive Parent, Abused/Abandoned Child and Angry/Impulsive Child modes have been shown to characterize borderline PD [11]. Furthermore, studies have found significant relations between schema modes and mental health disorders other than PDs, such as posttraumatic stress disorder, obsessive compulsive disorder, social anxiety disorder, depression, and eating disorders (see Bär et al. [13] for a review). Studies have also explored how schema modes relate to a range of other factors in mental health issues, for example patients’ qualitative perceptions of their own modes in eating disorders [17], behavioral impulsivity in patients with borderline PD with and without addiction [18], and network interactions between symptoms and schema modes in burnout [19]. Longitudinal studies show that schema modes predict future symptomatology, for example in PDs [20–23] and suicidality [24]. Finally, two studies found schema modes to mediate PD symptoms [25] and needs satisfaction in older adults [26]. 

Although these studies provide valuable insights about whether and how schema modes are related to different disorders and symptoms, they provide very limited information on the details of how schema modes operate. To test the central premise that mode activation occurs upon schema-related triggers, and what exactly triggers a schema mode, experimental research is needed. Activating schema modes under controlled circumstances will also allow us to explore and test how schema modes may bias information processing (e.g., in attention, interpretation, and memory) and behavior (e.g., automatic approach or avoidance tendencies). This will further our understanding of how schema modes contribute to the persistence of psychopathology. In this article we will first review the existing experimental literature on schema modes. Then, we will discuss experimental paradigms that could serve as mode inductions allowing for causal research. We end with methodological guidelines and a research agenda for experimental research on schema modes.

## State of the Art: Schema Mode Induction Procedures

The experimental literature on schema mode inductions appears to be rather limited. We conducted an extensive literature search for papers published from 2005 to 2024 on Google Scholar, PubMed, and PubPsych on the 13th of June 2024. During our search we used combinations of the following keywords: “schema modes”, “experimental induction”, “mode induction”, “schema therapy”, “evoking emotional states”, “inducing emotional states”, “experimental research”, “induced mood”, “emotion induction”, “mode activation”, “schema activation”, “manipulation”, “induced anger”, “induced vulnerability”. We identified five studies that used a stress induction interview, film clips, or art/drama therapy protocols to induce (emotional) states of different schema modes in PD samples. The study details are outlined in Table 1.

### Stress Induction Interview

Using a stress induction interview to elicit an anger response (based on Dimsdale et al. [27]), borderline, antisocial, and cluster C PD patients as well as non-patient controls were asked to recall and describe a past conflict with an aggressor [28]. An abbreviated version of the Schema Mode Inventory (SMI; Young et al. [29]) was used to assess changes in participants’ anger-related schema modes (Angry Child, Enraged Child, and Bully and Attack mode). Overall, the induction led to a significant increase in the three anger-related modes from baseline to post-interview. Specifically, the Angry and Enraged Child modes were significantly increased within the borderline PD and cluster C PD groups, whereas the Bully & Attack mode increased significantly only in the cluster C PD group. The Angry Child mode increased to a significantly greater extent in the borderline PD group than in the other groups. The non-patient control group demonstrated a significantly smaller increase in the Bully & Attack mode compared to the other groups.

## Film Clips

Two studies used abuse-related film clips to induce schema modes [11, 30]. The scenes depicted physical, emotional, and sexual abuse in scenes from the commercial movie “No Child of Mine” [31]. One study [11] included patients with borderline PD, cluster C PDs, and non-patient control participants. Schema mode activation was assessed with a state-version of a predecessor of the Schema Mode Inventory self-report questionnaire (SMI; Young et al. [29]). Participants viewed the 10-minute abuse-related clips or a neutral film clip in counterbalanced order with one week in between viewing sessions. Across groups, the abuse-related film clips led to significantly higher increases in Abandoned/Abused Child, Punishing Parent, and Detached Protector mode compared to the neutral clip. The borderline PD group demonstrated a significantly greater increase in the Detached Protector mode than the other groups following the abuse-related film clips (but not following the neutral clip). In contrast, the cluster C PD group demonstrated a significant decrease in the Detached Protector mode after the abuse-related film clip compared to the other groups [11]. The second study [30] used the same procedure but with a longer film clip of 20 min and an additional group of patients with antisocial PD. The abuse-related film clip led to a significant increase in a range of maladaptive modes and a significant decrease in the Healthy Adult and Happy Child modes in the borderline PD group only [30].

## Techniques from Art and Drama Therapy

Two studies induced schema modes using techniques from art and drama therapy [32, 33]. One study aimed to induce the Vulnerable Child and Angry Child modes in nine patients with mixed PD diagnoses at a forensic psychiatric center [32]. Participants received a drama therapy intervention of five sessions. In the second session, participants were asked to act out a typical family dinner based on their own past experiences, which was intended to activate meaningful emotions from childhood. In the third session, intended to induce feelings of vulnerability, participants were asked to act out a situation where their emotional needs were left unmet. During the fourth session, participants were asked to act out a recent situation that made them angry. Using the Mode Observation Scale (MOS; Bernstein et al., 2009), independent raters assessed schema mode activation minute-by-minute of three sessions that were specifically aimed at eliciting schema mode-relevant emotions. Schema mode activation before each intervention and the highest level of schema mode activation during each session were rated. The three sessions intended to induce schema-relevant emotions indeed led to a significant increase in participants’ Vulnerable Child mode (peak compared to before the intervention), although the overall peak levels were only low to moderate. None of the sessions, including the session explicitly aimed at eliciting anger, led to a significant increase in the Angry Child mode. The second study compared drama, art, and psychomotor therapy sessions intended to induce the Vulnerable Child mode in 20 adult male patients with mixed mental health diagnoses in a forensic psychiatric center [33]. In each type of therapy, the third session was specifically intended to elicit the Vulnerable Child mode. The fourth session was a regular intervention according to the specific therapy type. Patients rated vulnerability items of the SMI short form (SMI-R; Lobbestael et al. [34]) before and after sessions three and four. These sessions were videotaped and independently rated on Vulnerable Child mode activation (before each intervention and peak levels per session) by two of the therapists who provided the interventions using the MOS (Bernstein et al., 2009). The therapists did not rate their own sessions. According to their ratings, in all therapy types, both the Vulnerable Child session (session three) and the regular therapy session (session four) significantly increased the Vulnerable Child mode. In contrast, participants’ self-report ratings (SMI-R) did not show any statistically significant changes in Vulnerable Child mode activation in any of the sessions (Table 1).

**Table 1:** Studies using experimental schema mode manipulations

Study	Sample	Manipulation	Outcome Measures	Results
Lobbestael et al. (2009) [28]	35 healthy controls, 45 borderline PD, 21 antisocial PD, 46 cluster C PD	Autobiographical anger recall (stress induction interview)	SMI [29]: Angry Child, Enraged Child, Bully & Attack POMS [35]	Overall changes: ↑ All three anger-related modes Within group differences: ↑ Angry Child and Enraged Child in borderline and cluster C PD ↑ Bully & Attack in cluster C PD n.s. in antisocial PD Between group contrasts: ↑ Angry Child largest in borderline PD ↑ Bully & Attack significantly smaller in healthy controls
Arntz et al. (2005) [11]	18 healthy controls, 18 borderline PD, 18 cluster C PD All female patients	10-minute abuse-related film clip (‘‘No child of mine’’ [31])	Predecessor of SMI [29]: Detached Protector, Punishing Parent, Angry Child, Abandoned/Abused Child, Compliant Surrenderer, Overcompensator, Bully & Attack, Healthy Adult	Overall changes: ↑ Abandoned/Abused Child, Punishing Parent, Detached Protector (across groups) Between group contrasts: ↑ Detached Protector largest in borderline PD ↓ Detached Protector in cluster C PD
Lobbestael & Arntz (2010) [30]	Same sample as Lobbestael et al. (2009) 35 healthy controls, 45 borderline PD, 21 antisocial PD, 46 cluster C PD	20-minute abuse-related film clip (‘‘No child of mine’’ [31])	Abbreviated version of the SMI [29]: Maladaptive modes^a^, Healthy Adult, Happy Child POMS [35]	Within group differences: ↑ Maladaptive modes in borderline PD ↓ Healthy Adult, Happy Child in borderline PD Between group contrasts: ↑ Maladaptive modes largest in borderline PD n.s. Healthy Adult and Happy Child decrease
Keulen-de Vos et al. (2017) [32]	4 antisocial PD, 4 borderline PD, 1 antisocial + borderline PD Forensic male patients	Three role play interventions	Independent rater MOS: Vulnerable Child, Angry Child	Within group differences: ↑ Vulnerable Child across patients in all three interventions Low/moderate peak levels of Vulnerable Child activation n.s. Angry Child changes
Van den Broek et al. (2021) [33]	15 substance use disorder patients, 12 non-specific PD, 7 antisocial PD, 4 paraphilic disorder patients Forensic male patients	Three types of therapy, each including two role play interventions Each patient received one type of therapy: drama therapy (*n* = 8) art therapy (*n* = 6), psychomotor therapy (*n* = 6)	Therapist-rated MOS: Vulnerable Child SMI-R (self-report): Vulnerable Child	Within group changes: ↑ Vulnerable Child across the two interventions in all therapy types (MOS) n.s. SMI-R changes

PD = Personality Disorder; n.s. = non-significant; Schema Mode Inventory (SMI; Young et al., 2007); Profile of Mood States (POMS; McNair et al., 1992); Mode Observation Scale (MOS; Bernstein et al., 2009); Schema Mode Inventory short form (SMI-R; Lobbestael et al. [34]). ^a^ = Vulnerable Child, Angry Child, Enraged Child, Impulsive Child, Undisciplined Child, Compliant Surrenderer, Detached Protector, Detached Self Soother, Self Aggrandizer, Bully & Attack, Punishing Parent, Demanding Parent

## Summary and Methodological Implications

Research on schema mode activation has been sparse, and we only identified five highly heterogenous studies during our literature search on schema mode inductions. These studies used different induction procedures (stress interview, film clips, and art/drama therapy), intended to trigger different mode-related emotions and feelings (anger, different types of distress, vulnerability) in different populations (borderline PD, antisocial PD, cluster C PD, forensic patients, healthy controls), using outcome measures of self-report and observations of schema mode activation (SMI, MOS). Most procedures resulted in schema mode activation to at least some extent. Three methodological observations are noteworthy here:

(1) Each procedure resulted in different patterns of schema mode activation depending on the participant samples. For example, the stress interview induced the Bully & Attack mode in patients with cluster C PD but not in patients with borderline PD. Differential patterns were found in most of the studies. This implies that these experimental procedures do not inherently carry a trigger for one specific schema mode, but rather that they interact with participant characteristics to result in various schema modes potentially being activated. Methodologically, this means that an experimental procedure that successfully induced one or more schema modes in a certain patient group may not result in the same schema modes being activated when applied to a different patient group. Thus, it will be crucial to validate the same procedure for different clinical populations.

(2) The effects induced by the procedures do not appear to be very robust or specific. For example, the findings in the cluster C PD group in Arntz et al. [11] did not replicate to Lobbestael and Arntz [30] although comparable procedures (i.e., clips of the same abuse-related movie) and schema mode measures (variations of the SMI) were used. Additionally, in most of the studies, the procedure induced multiple schema modes within individual participants. This points to the need for test-retest reliability data of each experimental procedure.

(3) Ratings by therapists and patients do not necessarily align (e.g., Van den Broek et al. [33]). It is possible that therapists and patients interpret schema mode activation differently, and the discrepancy could also be explained by the confounding use of different schema mode measures (i.e., MOS and SMI). However, in our own research (Hoogendoorn et al., in prep.; see 10.17605/OSF.IO/UPKC7 for pre-registration) we also observed that therapists’ ratings did not completely align with patients’ ratings even when using the same ratings scales. In both the Van den Broek et al. [33] study and our recently completed study (Hoogendoorn et al., in prep), therapists ratings showed significant effects whereas patient ratings did not. At this point it is not possible to confidently explain this discrepancy, as there are different options that have not been ruled out yet. For example, therapists may be better trained and more accurate in identifying schema mode activation than their patients, or perhaps that therapists are biased towards seeing more schema activation and attributing patient distress to schema modes. In any case, the methodological implication is that ratings of therapists and patients cannot be used interchangeably, even when using the same instrument.

### Emotion Induction Paradigms

The studies on schema mode activation that were reviewed above made use of procedures that intended to induce schema mode activation by eliciting schema-relevant emotions. There is a rich body of literature dedicated to the design and validation of experimental procedures to elicit emotions. We gratefully make use of this literature for moving the research on schema modes forward. Several reviews and meta-analyses provide information about which stimuli are most potent to elicit specific emotions or categories of emotions. Siedlecka and Denson [36] organized experimental procedures into visual stimuli, music, autobiographical recall, situational procedures, and imagery procedures. They conclude that visual stimuli (pictures, videos) appear to be most effective in eliciting the basic emotional states of anger, disgust, surprise, happiness, fear and sadness. Music was found to effectively elicit happiness, fear and sadness. A meta-analysis by Fernández-Aguilar et al. [37] that included 45 studies showed significant differential effects of neutral, positive and negative film clips on self-reported valence and arousal. In both articles, the authors concluded that there was inconsistent and little evidence that combining stimuli (e.g., pictures with music) increased emotional effects compared to singular inductions. Another second review, Joseph et al. [38] additionally included self-referential statements (Velten statements), facial and bodily postures, odor, and situational procedures (i.e., receiving predetermined feedback on a bogus test, coping with a challenging situation, and social pressure situations). They concluded that most stimuli were effective in eliciting emotions, but the strongest stimuli (i.e., film clips with instructions) were eight times more effective than the weakest stimuli (i.e., cartoons). This review provides further methodological recommendations for selecting emotional stimuli, which we believe are also relevant for selecting stimuli for schema mode activation:

(i) Consult existing databases when selecting your experimental stimuli. These databases provide rich sets of validated stimuli along with information about their impact (e.g., valence, arousal, type of emotion). We refer the reader to Table 2 for an overview of existing resources, including the described reviews and meta-analysis, a review specifically on emotional effects of virtual reality, and databases with film clips.
(ii) Select your experimental stimuli in line with the cognitive (e.g., abandonment) and/or emotional theme (e.g., sadness) consistent with the schema mode you aim to induce.
(iii) If you need to combine stimuli to cover all relevant mode characteristics, do not assume that the stimuli effects automatically add up. Make sure to pilot test new combinations first.
(iv) When possible, be transparent about the goal of the trigger instead of using deceit (e.g., a cover story). Effectiveness of stimuli appeared to be higher without deception. We suggest including demand checks when possible. Of course, sometimes deception is necessary to prevent demand effects.

**Table 2:** Resources for emotional stimuli

Source type	Content	Description
Reviews	General	Siedlecka and Denson [36]: classification of 427 articles in five categories: visual stimuli, music, autobiographical recall, situational procedures, imagery. Reported impact on self-reported emotion and physiological measures for anger, disgust, surprise, happiness, fear and sadness. Joseph et al. [38]: classification of 529 articles in 11 categories: Velten statements, imagery, autobiographical recall, film, story, music, pictures, feedback, coping challenge, face/body posture, cartoons, odor, social pressure. Reported impact on positive and negative affect.
	Virtual Reality	Diniz Bernardo et al. [39]: review of 61 articles on virtual reality effects on emotions (joy/happiness, fear, sadness, disgust, anger, relaxation, stress and anxiety) with self-report and physiological ratings.
Databases of visual stimuli	Database of Emotional Videos from Ottowa (DEVO); Ack Baraly et al. [40]	291 brief obscure neutral, positive and negative film clips with a variety of themes (e.g., social interactions, nature) with arousal and impact ratings.
	Film catalogue for emotion induction; Gilman et al. [41]	List of film clips to elicit amusement, anger, disgust, fear, happiness, joy, neutral state, sadness, and surprise. Includes self-report affect ratings and physiological and facial ratings.
	LIRIS-ACCEDE video database; Baveye [42]	9.800 film clips from 160 freely available movies ranked according to valence and arousal ratings.
	Standardized verbal and non-verbal film clips; Jenkins and Andrewes [43]	Overview of 60 film clips to elicit amusement, anger, disgust, fear, happiness, sadness or neutral state, defined in age groups (18–45 years and 46 + years) with an index rating of emotion specificity.
	International Affective Picture System (IAPS); Lang et al. [44]	Well-known picture database including more than 1.000 pictures with a wide range of themes, rated on valence, arousal, and dominance by males and females separately.
	Faces databases	See for an overview of existing face databases https://libguides.princeton.edu/facedatabases#s-lg-box-27690615

## Two Procedures for Experimental Schema Mode Induction

Here we provide two procedures that we deem suitable for experimental schema mode induction: guided schema mode procedures and using experimental triggers to activate schema modes. These procedures are informed by the five studies on schema mode activation and the emotion induction paradigms reviewed earlier. Below, we explain both procedures and illustrate them with two examples for each procedure and outline their advantages and disadvantages. We finish this section with final general recommendations.

### Procedure 1: Guided Schema Mode Induction

Two types of guiding procedures are particularly useful for guiding participants into the experience of a prescribed schema mode: (1) autobiographical recall and (2) scripted imagery. Autobiographical recall and (to a lesser extent) scripted imagery reliably elicit anger, fear, disgust, happiness and sadness reflected by self-report and physiological measures [36, 38]. In the autobiographical recall procedure, participants are asked to recall a specific memory from their life that elicits the target emotion. In scripted imagery, the participant is instructed to imagine a specific scenario (that the participant has not necessarily experienced personally) to induce an emotional state. Both procedures are suitable for research questions where the schema mode is prescribed to the participant (i.e., independent variable). These guided procedures are less suitable for research questions about possible triggers of mode activation, i.e., where schema mode activation is the dependent variable or mediator. When using autobiographical recall or scripted imagery, we recommend using a memory or imagined scenario that covers both the emotional and the cognitive theme of the target schema mode.

### Examples of an Autobiographical Recall and Scripted Imagery Procedure for Guided Schema Mode Induction

#### Autobiographical Recall 

We outline an example of what an autobiographical recall procedure for inducing the Vulnerable Child mode might look like, with overwhelming distress as the emotional theme and helplessness as the cognitive theme:

The participant is asked to close their eyes and sit comfortably. They receive a prompt for autobiographical recall containing a description of the relevant aspects of the schema mode, in this case: “Please think back to a specific moment in your life that you *[felt very anxious*,* sad*,* overwhelmed and helpless]*”. The participant is instructed to recall this memory as vividly and with as much detail as possible, and to relay it to the experimenter in the present tense and from the first-person perspective, as if it is happening right here and now, e.g., “I am in my room, curled up on the bed and sobbing. We just had a huge fight. I see the pale green walls, hear rain ticking on the window, and I feel the softness of the sheets on the bed. My body is shivering… I feel so alone and desperate. I am panicking and don’t know what to do.”

#### Scripted Imagery Example

The procedure for scripted imagery looks similar, but instead of recalling a memory, the participant is asked to imagine a specific scenario. For example, to induce the Angry Child mode, the participant may read a story in which they were in an important meeting but being constantly interrupted and ignored (cognitive theme) and feeling very angry about it (emotional theme). They read the story and then close their eyes and imagine themselves in this situation as vividly and with as much detail as possible, relaying this image to the experimenter in the present tense and from the first-person perspective. More guidance from the experimenter is typically needed in scripted imagery to make the image vivid (e.g., by instructing the participant to focus on bodily sensations and sensory details of the image) [36].

Depending on what the researcher is interested in, the participant can either be instructed to keep the memory or the scenario in mind as vividly as possibly while completing measures that are not too distracting (e.g., verbally answering a few questions on brief single-item self-report ratings, or sitting still with continued physiological measures), or be instructed to let the memory/image drift away while holding on to their internal state “of [*being overwhelmed by intense anxiety*,* sadness and helplessness*]” when opening their eyes. They can then be asked to engage in other tasks (e.g., a computer task, or an interaction with a confederate in the lab) while keeping the internal experience of the schema mode activated.

### Challenges and Recommendations When Guiding Participants Into Schema Modes

Guided procedures come with specific challenges. First, participants may have trouble identifying a memory. Providing a starting point such as asking for a more recent experience covering the same theme or using the affect bridge technique (e.g. Arntz [45]) can help in selecting a relevant memory. Specific prompts can help if the participant can point to a general memory but has trouble coming up with a specific memory (e.g., asking for a specific time and place, e.g. Raes et al. [46]). Second, the experimenter should check whether the theme of the memory matches the target schema mode and whether the participant is indeed experiencing that mode. For example, the Vulnerable Child Mode relates to the Mistrust/Abuse schema, which would match a memory of an explosive fight with their partner, and when the participant describes feeling overwhelmed and helpless, this matches the experience of the Vulnerable Child mode. In a scripted imagery procedure this is less of a problem as the situation is prescribed to the participant and there is no need for the experimenter to check the theme as it is given. Third, participants may have trouble immersing in the memory or image. Using prompts to focus on sensory aspects or bodily sensations can help in that case [36]. For example, the experimenter can ask the participant to describe what they are seeing, hearing, smelling, which bodily sensations they are experiencing, and to look around in the image. It is important for immersion that the participant is continuously encouraged to describe the memory/image in detail, using the first-person perspective and in the present tense.

Based on these challenges, we first recommend that experimenters are well-trained in these procedures. Training would involve learning to assist participants into selecting a relevant and specific memory and creating a vivid image, continuously monitoring, and picking up on verbal and non-verbal cues about the level of immersion and providing prompts timely to avoid unnecessary distractions or interruptions. Second, we recommend screening for some threshold of the presence of the target schema mode in the participant sample, e.g., based on the Schema Mode Inventory [29] or its short version [34]. Most, if not all, people will be able to come up with a memory or image that matches the experimental instructions without experiencing noticeable levels of the target schema mode in their daily life. It is debatable whether the resulting state in these participants can be considered approaching a schema mode. Additionally, it may therefore be informative to check to what extent the induced target state is familiar to the participant (e.g., using a Likert scale).

### Advantages and Disadvantages of Guiding Procedures

The main advantage of a guided schema mode induction is its flexibility. The instructions can easily be adapted to match that of different schema modes while maintaining a uniform procedure which facilitates comparability and replicability of results. Moreover, the guiding procedure does not require any resources other than a thorough training of experimenters. The procedure also has disadvantages. First, memory recall is not suitable for all schema modes. For example, while the Vulnerable Child Mode (overwhelming emotions, helplessness) and the Demanding Critic Mode (unrealistically high standards of performance) have concrete and distinguishable elements, it is far more difficult to induce the more general and abstract Detached Protector mode (not feeling anything). Second, it is practically impossible to determine whether the memory recall or scripted imagery acts as a trigger for actual schema mode activation in real-time, or whether the mode is merely vividly recalled or imagined ‘as-if’ real. Finally, mental imagery is taxing on cognitive resources, and the vividness of imagery will fluctuate and dissipate over time [47]. This implies that opportunities for administering additional measures during or after the induction procedure is limited to brief and simple measures (or some physiological measures).

### Procedure 2: Experimental Triggers for Schema Mode Activation

The second procedure uses experimental triggers to activate schema modes. The main difference with the guiding procedure is that the experimenter provides an experimental stimulus to trigger the schema mode but does not prescribe the mode. The experimental trigger procedure allows for a detailed investigation on how and under what circumstances schema modes are activated (i.e., in which schema modes are the dependent variable or mediator). As with the guiding instructions, the trigger should match the cognitive and emotional theme of the target schema mode. Below, we describe two examples of experimental triggering procedures: using visual stimuli as triggers and a situational procedure.

### Examples of Using Visual Stimuli and a Situational Procedure To Experimentally Trigger Schema Mode Activation

#### Visual Stimuli Example

The studies by Arntz et al. [11] and Lobbestael and Arntz [30] were successful in triggering the Abandoned/Abused Child mode in patients with borderline PD using abuse-related film clips as experimental triggers. The theme of these film clips related to traumatic experiences often present in the history of patients with borderline PD and matched the cognitive and emotional theme of the Abandoned/Abused Child mode. For specific procedural instructions we refer the reader to the original publications. The databases mentioned in Table 2 provide a further resource for finding validated film clips with a wide range of cognitive and emotional themes.

#### Situational Procedure Example

Ongoing work by Peeters et al. (2023; stage 1 registered report; see https://osf.io/ng6vc/files/osfstorage for details) uses an adapted academic performance task (i.e., situational trigger) to induce the Demanding Critic mode. The performance and feedback element of the task matches the unrealistic high standards and perfectionism that is typical of this mode. Participants receive false feedback in which their performance on a simple math task appears to be decreasing slightly over time. Their emotional and cognitive responses are assessed and compared to an identical performance situation with neutral feedback to test for differences in Demanding Critic mode activation. Note that this procedure was extensively piloted first. Several other situational procedures have been validated in terms of emotional themes, please see the reviews by Siedlecka and Denson [36] and Joseph et al. [38] for an overview.

### Challenges and Recommendations When Using Experimental Triggers To Activate Schema Modes

The main challenge is that there is no validated set of experimental triggers for schema mode activation available yet. As in Peeters et al., (2023) extensive pilot testing will be required for each new study, and replication studies will be required as well. We recommend using a validated emotion induction procedures (see Table 2) as a starting point. As with the guiding procedure, it is recommended to screen the participants for presence of the target mode, for example with the relevant subscales of the SMI, as an inclusion criterion. In case mode presence is relatively low, the experimental trigger will likely not result in observable changes in emotion or schema mode activation. This may wrongly be interpreted as the experimental manipulation being invalid or ineffective.

### Advantages and Disadvantages of Experimental Triggers for Schema Mode Activation

Compared to a guiding procedure, using experimental triggers to activate schema modes has the advantage that participants may be kept naïve about the goal of the manipulation. This reduces the risk of socially desirable answers and demand effects. Moreover, schema mode activation is directly triggered and not ‘merely’ vividly recalled or imagined. The main disadvantage is that experimental triggers may inadvertently activate unintended modes as well. Note that the studies using a film clip of abuse (11, 30) induced a number of modes, including the Vulnerable Child mode, the Punishing Parent mode, as well as the Detached protector mode.

### General Recommendations for Experimental Schema Mode Induction Procedures

#### Control Groups

It is important to consider beforehand what type of sample (in terms of modes) would best fit the research question. Individuals who have a strong presence of the schema mode in daily life should respond more strongly to a relevant mode manipulation than individuals in which this mode is less markedly present. If such an interaction effect is observed, it suggests that the manipulation is not merely an emotional trigger but also addresses the intended schema mode. Experimental studies should therefore ideally include and compare groups of participants that significantly differ in the presence of the schema mode (e.g., based on SMI subscale scores). At the least, relevant SMI scores should be recorded for all participants included in the study.

### Control Conditions

An experimental control condition should be included that resembles the schema mode manipulation procedure as much as possible but without the specific mode theme. For example, a neutral or positive memory when using the guiding procedure with autobiographical recall, a neutral film clip when using visual stimuli, or - in case of a situational procedure - a version of the performance task where the predetermined feedback indicates an increase or no change in performance instead of a decrease. Ideally, a significant group x condition interaction is observed, where the experimental condition results in more schema mode activation in participants with a strong presence of the target mode than in participants low in the target mode, with no or only small group differences in the control condition.

### Manipulation Checks

Manipulation checks should be included to verify that the experimental procedure indeed activates the intended schema mode (reflected in a significant pre- to post-manipulation difference) compared to the control condition (reflected by a time x condition interaction). This requires assessing schema mode activation before and after each manipulation. Some of the studies reviewed in this paper used a state-adapted version of relevant SMI items (e.g., ‘[…] *at this moment*’) [11, 28, 30, 33]. This might be a convenient option. However, auto-correlation with the SMI scores used for screening or group allocation may lead to inflated effects. Another option would be to observe mode activation, for example using the MOS [48] or Likert ratings (cf., Hoogendoorn et al., in prep.; 10.17605/OSF.IO/UPKC7). Note, however, that these observational measures have not been sufficiently validated yet.

#### Post-Manipulation Assessments

In real life, schema mode activation may last for an extended period (e.g., hours or days). However, the duration of experimentally induced schema mode activation is likely much shorter (e.g., minutes). Memory recall and mental imagery are cognitively taxing, and participants will not be able to keep the emotional effects going for a long time. Situational procedures are generally less subject to cognitive depletion, but it is unlikely that these triggers are as impactful as real-life experiences (which would also be cause for ethical concerns). This means that post-manipulation assessments of other variables of interest (e.g., self-report measures of emotion states, physiological measures, computer tasks of cognitive biases) should be kept as brief as possible. Researchers may consider providing prompts or repeatedly present (parts of) the experimental trigger to boost or reactivate the mode. In any case, repeated manipulation checks throughout the experiment will give an indication of the duration of schema mode activation.

### Open Science Practices

We recommend that researchers make their pre-registrations, registered reports, pre-prints, open access publications, (pilot) datasets, codes and scripts, and experimental material freely available on open science platforms. This will help in collectively building a knowledge base on effective, valid and reliable schema mode induction procedures.

#### Research Agenda

Experimental research on schema modes in PD samples is needed to better understand how and under which circumstances schema mode activation occurs, and what the effects of schema mode activation on other relevant factors are. To answer these questions, it will be necessary to have the right tools. We concluded from our literature review that there is a lack of validated experimental procedures and reliable measures to induce and assess schema mode activation under controlled circumstances. The challenges that we identified for the two proposed experimental procedures (guiding and experimental triggers) suggest the same. Thus, developing and validating experimental procedures and reliable measures for schema mode activation are main priorities. We hope to kickstart research developments with the guidelines and recommendations outlined in this paper. In addition, with these methodological advances we will be able to study important research questions about the nature of schema modes in the future, which we consider central to the research agenda: How and under what circumstances are schema modes are triggered? And what is the effect of schema mode activation on other relevant factors?

#### Research Priority: Under What Circumstances Are Schema Modes Activated?

The most recent formulation of the theory underlying schema therapy is based on the formula ‘early maladaptive schema x coping style = schema mode’ and was proposed in a position paper by an international workgroup on schema mode concepts [10]. Specifically, a resignation coping style should result in a specific Child or Critic mode (e.g., Vulnerable Child or Demanding Critic), whereas an inversion coping style should result in specific Coping modes (e.g., Bully and Attack mode) depending on the early maladaptive schema (EMS) that is triggered. An avoidance coping style is proposed to result in more general coping modes regardless of the underlying schema, as the individual is expected to adopt a chronic and general avoidant response such as experiential avoidance in most aspects of daily life (e.g., Detached Protector mode). A complete list of specific predictions about which schema modes will be activated in response to which triggered schema and coping style combination has been published in appendix A of the position paper [10]. Whilst these predictions still await empirical testing, observational research can be conducted to get a sense of whether modes indeed co-occur with their predicted schema and coping style combination. For this, a large dataset will be required that includes cases with a variety of combinations of EMS and coping styles. Preliminary data on this have been collected and appear to be in line with predictions (Rijkeboer & Lobbestael, in preparation).

Experimental tests will subsequently be needed to verify the causality of interaction. This will require systematic research on experimental triggers of schema modes in (quasi-) experimental designs^1^ for which our methodological guidelines can be useful. For example, a research goal may be to validate triggers of the Self-aggrandizer mode. Based on the overview in the position paper [10] we predict that we need to trigger the Defectiveness & Shame schema in individuals with an inversion coping style. If we want to test the role of the coping style, for example, we may want to compare them to participants with the same Defectiveness & Shame schema but with an avoidant coping style instead. Participants may be guided with an autobiographical recall procedure where they recall a memory that involved high levels of shame (emotional theme) and defectiveness (cognitive theme) (see Fink-Lamotte et al. [49]), or the researcher can provide bogus embarrassing or negative performance feedback in a situational procedure (see Jamil & Llera [50]). The Self-aggrandizer mode may be assessed by administering the relevant state-adapted SMI items before and after the experimental manipulation and a control condition. These types of studies will help us test some of the central hypotheses of the theory underlying schema therapy more directly and inform us about the conditions under which specific schema modes are triggered.

#### Research Question: What Are Subsequent Effects of Schema Mode Activation?

Schema modes may maintain PD patients’ (interpersonal) problems because they influence how the individual engages with others and the world around them in a negative way. This leads to the research question of what the subsequent effects are of schema mode activation. Schema mode activation may distort information processing and schema- or schema mode-congruent information could be given priority over disconfirming information. This way the schema mode would contribute to a self-fulfilling prophecy and thereby maintain the PD. Such knowledge would help to better understand the chronicity of PDs and the causal role of schema modes in their maintenance. The most studied information processing biases are those of attention, interpretation and memory. There are validated procedures to assess such biases (see, Zhang et al. [51] for example measures, and Lobbestael & Arntz [52], for an overview of biases found to relate to PDs) although none of them have been adapted yet to schema modes specifically. Furthermore, schema modes may also drive automatic (avoidance or approach) behavior (assessed with, for example, an Approach Avoidance Task; Heuer et al. [53]) or different social behaviors (assessed with, for example, the Trier Social Stress Test; Kirschbaum et al. [54]), or a Prisoners Dilemma game. For example, in the ongoing work of Peeters et al. (2023), attention, interpretation, and memory bias towards performance-related information is tested with a scrambled sentences test (adapted from Sanchez-Lopez et al. [55]) and a self-referential encoding task (adapted from Derry & Kuiper [56]) immediately after the experimental procedure intended to activate the Demanding Critic mode. Similar studies will help us understand better what schema modes do and what their (causal or mediating) role is in PDs and other chronic psychological symptoms.

### Summary and General Conclusions

We started this paper by underlining the value of experimental research on schema modes. We then discussed the few studies that aimed to induce schema mode activation in PD samples. These studies provided information on useful methodological paradigms but also pointed to the lack of validated procedures and reliable measures. To stimulate further research, we included references to resources and formulated guidelines and recommendations for experimental research on schema mode activation. These included guiding participants into modes using autobiographical memory recall or scripted imagery and activating schema modes using experimental triggers. We explained for what type of research question each procedure could be suitable and discussed their advantages and limitations. In the research agenda, we emphasized that developing and validating experimental procedures and measures of schema mode activation is a priority, and we identified two important future research questions: (1) under what circumstances are schema modes activated? and (2) what effects does schema mode activation have on other relevant factors such as information processing and behavioral tendencies? Answering these questions will help validate the theoretical principles of schema therapy and provide more insight on the role of schema modes in the chronicity of PDs and other chronic psychological symptoms. Our intention by providing these priorities and guidelines is to fuel further open science dedicated to unravelling the important concept of schema modes.

## Key References

- Young, J. E., Klosko, J. S., & Weishaar, M. E. (2003). Schema Therapy. Guilford Press.
  - ○ This book presents the first conceptual overview of schema therapy for clinicians, detailing maladaptive schemas and the development of the approach.
- Giesen-Bloo, J., Van Dyck, R., Spinhoven, P., Van Tilburg, W., Dirksen, C., Van Asselt, T.,... & Arntz, A. (2006). Outpatient psychotherapy for borderline personality disorder: randomized trial of schema-focused therapy vs transference-focused psychotherapy. Archives of general psychiatry, 63(6), 649-658. https://doi.org/10.1001/archpsyc.63.6.649
  - ○ A comparison of schema-focused therapy and transference-focused psychotherapy in reducing disorder-specific symptoms and general dysfunction among patients with borderline personality disorder in community mental health centers.
- Arntz, A., Rijkeboer, M., Chan, E., Fassbinder, E., Karaosmanoglu, A., Lee, C. W.,& Panzeri, M. (2021). Towards a reformulated theory underlying schema therapy: Position paper of an international workgroup. Cognitive Therapy and Research, 1-14. https://doi.org/10.1007/s10608-021-10209-5.
  - ○ This article presents an extensive redevelopment of the schema mode taxonomy, incorporating recent research and the informed opinions of an international workgroup, including the relabeling of coping strategies and the introduction of new schemas and modes.
- Arntz, A., Klokman, J., & Sieswerda, S. (2005). An experimental test of the schema mode model of borderline personality disorder. Journal of Behavior Therapy and Experimental Psychiatry, 36(3), 226-239. https://doi.org/10.1016/j.jbtep.2005.05.005.
  - ○ This study tests the schema mode model of borderline personality disorder by assessing mode activation in response to an abuse-related film fragment in borderline personality disorder patients, cluster C personality disorder patients, and non-patient controls.
- Lobbestael, J., Arntz, A., Cima, M., & Chakhssi, F. (2009). Effects of induced anger in patients with antisocial personality disorder. Psychological medicine, 39(4), 557-568. https://doi.org/10.1017/S0033291708005102.
  - ○ This study examines the effects of an anger-induction interview on schema mode activation in borderline personality disorder patients, antisocial personality disorder patients, cluster C personality disorder patients, and non-patient controls.
- Young, J. E., Arntz, A., Atkinson, T., Lobbestael, J., Weishaar, M. E., van Vreeswijk, M. F. and Klokman, J. (2007). The Schema Mode Inventory. New York: Schema Therapy Institute. http://www.schematherapy.com/id49.htm.
  - ○ Introduction of the Schema Mode Inventory (SMI), a 124-item measure of emotional states and respective coping responses grounded in Schema-Focused Therapy.
- Lobbestael, J., & Arntz, A. (2010). Emotional, cognitive and physiological correlates of abuse-related stress in borderline and antisocial personality disorder. Behaviour research and therapy, 48(2), 116-124. https://doi.org/10.1016/j.brat.2009.09.015.
  - ○ This study investigated emotional, cognitive, and physiological responses to an abuse-related film fragment in borderline, antisocial, and cluster C personality disorder patients, as well as non-patient controls.
- Keulen-de Vos, M., van den Broek, E. P., Bernstein, D. P., Vallentin, R., & Arntz, A. (2017). Evoking emotional states in personality disordered offenders: An experimental pilot study of experiential drama therapy techniques. The Arts in Psychotherapy, 53, 80-88. https://doi.org/10.1016/j.aip.2017.01.003.
  - ○ This study evaluates the effectiveness of three drama therapy interventions in inducing vulnerability and anger in a small sample of forensic patients with antisocial and/or borderline personality disorder.
- Van den Broek, E. P., Strijbos, N., Vromen, J., van Duursen, S., Cousijn, J., Bosschaert, L., ... & Keulen-de Vos, M. (2021). A pilot study of arts therapy techniques to evoke emotional states in forensic patients. The Arts in Psychotherapy, 74, 101798. https://doi.org/10.1016/j.aip.2021.101798.
  - ○ This study explores the effectiveness of drama therapy, art therapy, and psychomotor therapy in inducing the Vulnerable Child mode in adult male forensic patients with substance-related, paraphilic, antisocial, or other personality disorders.

## Data Availability

No datasets were generated or analysed during the current study.

## Footnotes

1 We assume here that coping styles are trait characteristics and cannot be experimentally manipulated.
